# Strategies to improve COVID-19 vaccination coverage in Manyara region, Tanzania, July to September 2022: best practices and lessons learned

**DOI:** 10.11604/pamj.supp.2023.45.1.39608

**Published:** 2023-06-08

**Authors:** Violet Mathenge, Chima Onuekwe, Shafique Nass, Caroline Akim, Erick Msunyaro, Elirehema Mfinanga, William Pascal Mambo, Suten Geofrey Mwabulambo, Suleiman Manozas, Damas Kayera, Florian Tinuga, Sisay Tegegne, William Mwengee, Phionah Atuhebwe, Yoti Zabulon

**Affiliations:** 1 World Health Organization, Tanzania Country Office, Dar es Salaam, Tanzania,; 2 World Health Organization, Inter-Country Support Team - East and Southern Africa, Harare, Zimbabwe,; 3 Health Promotion Section, Ministry of Health, Dodoma, Tanzania,; 4 Tanzania Medicines and Medical Devices Authority, Dodoma, Tanzania,; 5 Office of the Regional Commissioner, Manyara Region, Babati, Tanzania,; 6 World Health Organization, Regional Office for Africa, Brazzaville, Congo

**Keywords:** COVID-19, vaccination, pandemic, vaccination coverage, Tanzania

## Introduction

The introduction of COVID-19 vaccines was a game changer in the evolution of the pandemic at a time when countries experienced a marked loss of lives coupled with overstretching of already fragile health systems [1]. By the end of 2021, the global mortality had reached five million [2,3]. The pandemic negatively impacted all essential health services, with the most significant disruption noted in lower-income countries [4]. The rollout of COVID-19 vaccines cast a ray of hope to reopen economies and see an end to the pandemic. The rapid evolution of the outbreak and the risk of emerging new, more virulent variants further underscored the urgency for vaccine rollout [5]. Despite laudable progress in the expedited development of vaccines and efforts to improve equitable distribution, vaccination rates remain low in African countries compared to the rest of the world [6]. As of November 2022, only 32% of the African population had received at least one dose of vaccine, compared to the global average of 69% [2,3]. Across the African continent, multiple barriers exist to vaccine accessibility, misinformation, fear, lack of trust in vaccines, low-risk perception, and systemic and operational obstacles [7-9].

Low-risk perception and vaccine hesitancy was particularly evident in Tanzania, where a year following the launch of COVID-19 vaccination in the country, the national coverage for the eligible population remained below 8% [10]. Manyara region in Northern Tanzania was the poorest performing region in the country, with vaccination coverage standing at 3.7%. Of the 12 vaccines with WHO Emergency Use Listing (EUL), five were approved for use in Tanzania by the Tanzania Medicines and Medical Devices Authority (TMDA) [11,12]. The Government of Tanzania, therefore, tasked partners to provide technical and financial support to enable regions to implement intensified vaccination campaigns in June and July 2022. In collaboration with the Regional Health Management Team (RHMT) and partners, WHO provided technical support to Manyara region and supported and coordinated the pre, intra, and post-campaign phases. This support was replicated in September 2022 as it was still among the three least-performing regions for COVID-19 vaccination, with a vaccination coverage of 40%. As African countries intensify efforts to improve vaccination coverage towards the global vaccination targets, the importance of learning lessons from country experiences comes to the fore [13]. Therefore, this paper aims to document best practices and lessons learnt in adapting high-impact vaccine delivery strategies to increase COVID-19 vaccination coverage in Manyara region, Tanzania.

## Methods

**Study setting:** two COVID-19 vaccination campaigns were conducted in July and September 2022 in Manyara region, one of Tanzania mainland’s 26 administrative regions. The region comprises five districts with seven councils: Babati Town, Babati, Hanang´, Mbulu Town, Mbulu, Simanjiro and Kiteto Councils, with 27 divisions, 142 wards, 533 villages and 1945 hamlets. Manyara has a population of 1.9 million and a population density of 43 people per square kilometre, based on the census conducted in August 2022 [14]. The population density is highest in Babati, Mbulu and Hanang´ councils, while Kiteto and Simanjiro are sparsely populated and have predominantly nomadic communities. The region is served by 240 health facilities, with 202 offering COVID-19 vaccination and routine immunisation services. COVID-19 vaccination in the region is coordinated at the Regional Health Management Team (RHMT) offices in Babati Town Council.

**Study population:** the study population comprised all individuals aged 18 years and above in the region, the eligible population for vaccination in the region according to the National Vaccine Deployment Plan (NVDP). The total population above 18 years across the seven councils was 956,452. Adults with cormobidities, health care workers, individuals aged 45 years and above and essential frontline workers were classified as the priority target population. All individuals aged below 18 years of age were excluded from the study.

### Interventions

**COVID-19 vaccination campaigns:** we conducted two mass vaccination campaigns in July and September 2022. World Health Organization provided technical guidance to the region in adapting and contextualising the standard operating procedures (SOPs) for the campaigns and coordinated campaign activities. Various strategies were applied in the planning, implementation and post-implementation phases as follows:

### Pre-implementation

**Micro-planning:** in the planning phase, critical elements of the vaccination campaigns were captured in a comprehensive regional microplan by regional and district/council health management teams with technical support from WHO. A readiness assessment checklist based on the micro-plan was administered to all districts prior to the campaigns to assess their preparedness level and address any bottlenecks. Each ward and team’s micro-plan was verified, and a copy was kept at the regional level for reference before implementation.

**Advocacy and community engagement:** in July 2022, a regional advocacy meeting presided over by the regional Commissioner was held to improve coverage. Similarly, the Council Health Management Team (CHMT) supported by WHO made specific performance evaluation presentations at the district levels. Advocacy to regional and council leadership was repeated during the September campaign and was again followed by community mobilisation through community-level leadership. The RHMT, CHMT, and WHO engaged the Ward Executive Officers (WEO), District Executive Officers (DEO) and Village Executive Officers (VEO) for the community mobilisation process.

**Training:** with the technical support of WHO, 711 teams with 71 supervisors were trained before the implementation of the campaign. The training entailed both didactic and practical sessions and was conducted over a period of 5 days. Trainees were selected from among health workers and community members with a record of previous vaccination campaign experience. The training curriculum covered vaccination techniques, advocacy, risk communication and community engagement, and vaccine and data management, management of Adverse Events Following Immunization (AEFI) among other topics.

### Implementation

**Vaccination campaign strategies:** a mixed strategy approach was adopted to increase vaccine uptake and ensure that all eligible beneficiaries were reached. Thus, the vaccination strategies we employed included fixed posts at health facilities, mobile outreaches, events-based approaches (i.e. at markets, churches, bus terminals, mosques) and house-to-house.

**Campaign targets:** the vaccination target for the first campaign was to attain 40% coverage for the eligible population, in line with national targets. For the Manyara region, 40% coverage translated to vaccinating 213,434 eligible persons. For the September round, the region aimed at 70% coverage by the end of the exercise. To achieve this, 283,600 individuals should have completed the primary series of COVID-19 vaccines over 5 days. For both rounds, the campaign targets were disaggregated to district, health facility and vaccination team levels. For the first round, each vaccinating team was expected to vaccinate 50 individuals per day; for the second round, the target was set at 80 vaccinations per day. Vaccination coverage was calculated by dividing the number of individuals who had completed the primary series of vaccines by the total eligible population. Given that a single dose regimen was used during the campaign, vaccination coverage was calculated by dividing all individuals vaccinated by the eligible population.

**Vaccines administered:** during both campaigns, the Janssen Ad26.COV2.S vaccine was administered. This vaccine initially received the WHO Emergency Use Listing (EUL) as a single intramuscular dose of 0.5ml after the initial Phase 3 trial. The current WHO recommendation is the administration of two doses 2- 6 months apart, with consideration for a single dose for hard-to-reach populations and in cases of supply or delivery limitations [15]. As per the Tanzania National Vaccine Deployment Plan, a single intramuscular dose schedule for the Janssen Ad26.COV2.S vaccine was adopted. Hence, all individuals who received a single dose were considered to have completed the primary series. Moreover, two of the seven councils have predominantly nomadic populations. Therefore, the programmatic challenges associated with reaching these populations justified using a single-dose regimen.

**Storage and cold chain capacity:** Manyara region had three vaccine supply chain management levels: the regional, district and health facility. Vaccines were distributed and redistributed during the campaigns at all levels based on cold chain capacity, vaccine stocks and potential expiry to minimise wastage. The region had a net refrigeration (+2 to +8°C) capacity of 135 litres, catering for COVID-19 vaccination and routine immunisation, and zero ultra-cold freezers. One Regional Vaccine Store (RVS) supplied seven District Vaccine Stores (DVS). The DVS are equipped with 2-3 ice-lined refrigerators and freezers and supply 202 health facilities. Immunising health facilities had either solar, electricity or electricity and Liquefied Petroleum Gas (LPG) powered refrigerators. Remote temperature monitoring devices and non-remote devices (i.e., fridge-tag devices (FT2) that monitor routine, and COVID-19 vaccines had been installed at all levels.

**Team composition:** each vaccinating team comprised a vaccinator, recorder and community mobiliser. Across 533 villages, 771 teams conducted the 9-day campaign in July 2022, while 703 teams conducted the 5-day campaign in September 2022.

**Monitoring and supportive supervision:** public health experts were deployed for technical coordination, risk communication and community engagement, finance and administration, data management, monitoring and evaluation to provide technical direction for result-oriented campaigns. A standardised electronic-based checklist under the Open Data Kit (ODK) platform was developed and used by supervisors to assess the quality of the interventions across the seven councils. Daily in-process monitoring was conducted during the campaigns with feedback on the status of indicators, coverage and gaps.

### Post-implementation

**Intra-action review:** following the campaigns, a COVID-19 vaccination intra-action review was carried out to identify best practices and critical actions to address challenges and institutionalise best practices.

**Data collection:** data were collected using standardised health facility registers, tally sheets and ledger books. The tools, completed by trained health workers, comprise sections on socio-demographics, past medical history and COVID-19 vaccination history. The data management team utilised the data on the three tools for triangulation purposes to increase the credibility of the data, the primary source of information being the facility registers. Data was subsequently transferred from all facility registers onto a detailed summary tool in Microsoft Excel. Data generated before the interventions were used as baseline data to inform resource allocation during the microplanning process.

**Data management and statistical analyses:** the data management team transferred the data from the paper-based tools onto an MS Excel spreadsheet used for data cleaning. Data cleaning included checks for missing values, inconsistent responses and duplicate observations. Cleaned data were then exported to STATA version 15.0 (College Station, Texas 77845 USA) for all analyses. New variables, such as vaccine coverage, were created and labelled appropriately. The data were collated and analysed by comparing vaccine coverage before and after implementing the interventions.

**Ethical considerations:** relevant data obtained from the RHMT were de-identified during processing to preserve confidentiality. Permission to use the data was obtained from the Ministry of Health, Tanzania. The data were stored in an external hard drive, and access was restricted to authorised individuals.

## Results

**Figure 1:**
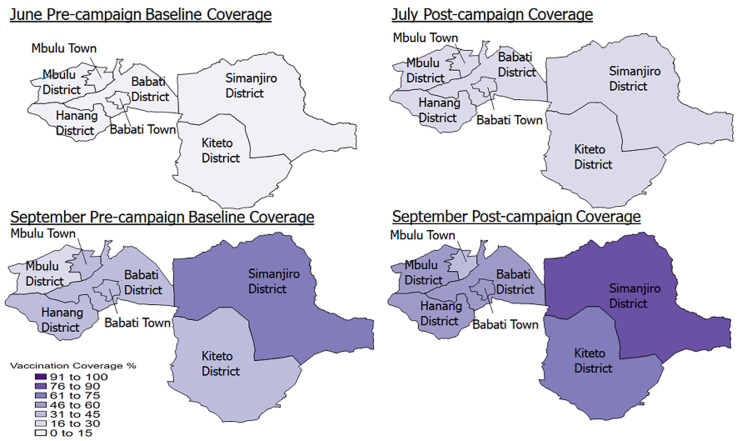
pre and post-campaign COVID-19 vaccination coverage June 2022 and September 2022, Manyara region

V**accination coverage:** the baseline vaccination coverage as of June 2022 at the regional level was 3.7% for Manyara region. At the council level, the coverage was highest in Mbulu (9%) and lowest in Simanjiro at 1%. Following the first campaign conducted in July, overall coverage at the regional level was at 20%, with Mbulu retaining the highest coverage (29%) and Hanang´ the lowest coverage at 18% (Figure 1). Following the September campaign round, the regional coverage was 59%. Simanjiro had the highest coverage (88%), followed by Babati TC (69%) and Kiteto (61%) councils. Mbulu recorded the lowest coverage at 43% (Figure 1).

**Performance against campaign targets:** during the July round, the region vaccinated 157,051 (74%) out of the targeted 213,434 individuals, while in September, 177,862 (63%) of the targeted 283,600 received the vaccine. At the district level, the performance (vaccinated/targeted) was as follows: Simanjiro DC 32,993/27,681 (119%), Babati DC 38,381/49,251 (78%), Hanang´ DC 28,213/40,954 (69%), Kiteto DC 24,138/36,120 (67%), Babati TC 8,253/14,286 (58%), Mbulu DC 16,139/28,646 (56%), Mbulu TC 8,934/16,496 (54%). In the second round, the performance by district was as follows: Babati TC 17,619/19,200 (92%), Kiteto DC 33,033/48,000 (69%), Simanjiro DC 24,085/36,000 (67%), Hanang´ DC 34,375/54,800 (63%), Mbulu DC 22,781/38,000 (60%), Babati DC 36,988/65,600 (56%) and Mbulu TC 8,981/22,000 (41%).

**Figure 2:**
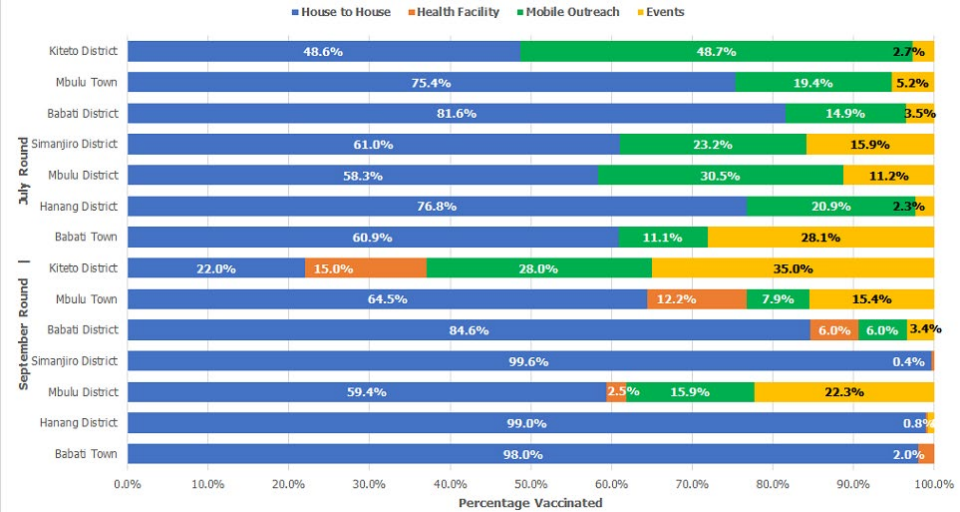
COVID-19 vaccination strategy by district, July compared to September 2022, Manyara region

**Vaccination strategy:** Figure 2 shows the percentage of individuals vaccinated using the various strategies, disaggregated by the district for the two rounds. In the July round, the majority (67%) of people vaccinated were reached through the house-to-house strategy. This was particularly the case in Babati and Hanang´ district councils, where the percentage reached through house-to-house was 82% in Babati and 77% Hanang´. In Kiteto district, >50% were reached through events and mobile outreach. In the September round, the vaccination teams reached 77% of the people through the house-to-house strategy. In Kiteto, less than 30% of vaccinations were conducted at households, with events and mobile outreach having a near-equal distribution of vaccinated numbers.

## Discussion

Manyara´s experience in improving vaccination coverage highlights the following lessons. First, using multiple vaccine delivery strategies emphasising the house-to-house strategy was effective. Second, setting campaign targets and using data to contextualise strategies coupled with comprehensive planning proved helpful. Indeed, mass campaigns were very effective in the scale-up of vaccine uptake in Manyara, as evidenced by the marked increase in vaccination coverage between July 2022 and September 2022 in all seven districts. Given the urgency to roll out vaccines, this strategy enabled the region to increase coverage quickly. During the first campaign, vaccination coverage increased fivefold within 9 days. Similarly, for the second campaign, a quarter of the target population was vaccinated within 5 days. Similar findings have been reported in several African countries, with 9 out of the 20 countries termed as a priority in the WHO African region (due to low COVID-19 vaccination coverage), recording an increase in doses administered following mass vaccination campaigns [16]. In Ghana, a 34% increase in doses administered following the introduction of mass campaigns in February 2022 was reported [17]. Likewise, in Niger and Zambia, nationwide campaigns conducted in October 2022 resulted in an additional 1.2 million and 1.9 million people completing the primary series, respectively [16].

These findings are in line with two previous surveys conducted across 29 African countries, which demonstrated the population’s willingness to be vaccinated with emphasis on preference for vaccination at convenient locations such as homes and offices and zero cost to the beneficiaries [17,18]. For both rounds, most vaccinated people were reached through the house-to-house strategy, which improved vaccine accessibility for hard-to-reach areas and created room for one-on-one sessions to counter misinformation and myths that drive hesitancy [18]. However, despite the undeniable gains from this approach, it is highly resource intensive due to the considerable number of teams required to cover large areas [19]. In addition, mass campaigns are associated with higher risks of adverse events following Immunisation (AEFI) arising from immunisation errors, and anxiety-related and coincidental reactions. While previous studies have reported low rates of AEFI, questions about adequate monitoring and reporting of AEFI in these settings arise [20] Given the mobile nature of this strategy, the recommended post-vaccination observation periods may not be fully observed, reducing the chances of detection [21]. During the vaccination campaigns, no reports of AEFI were received at the regional level. However, efforts to strengthen AEFI surveillance in all regions are ongoing.

Regional and council-level advocacy and leveraging existing community structures and capacities was vital. Detailed discussions on regional performance against the rest of the country challenged leadership and cultivated ownership at all levels. Moreover, the involvement of local leaders and influencers at the grassroots, as opposed to the initial strategies of using community health workers (CHW) that were not known in the communities, was vital. This observation has been previously reported; while the role of CHWs is irrefutable, a whole societal approach rather than a singular investment in CHWs is more effective [22]. This was notably evident in Mbulu DC, during the second round, where a special Primary Health Care Committee (PHC) meeting under the leadership of the District Commissioner (DC) alleviated hesitancy. The study showed that target setting for the region and disaggregation of targets down to the individual level made the campaigns very result oriented and was a critical component during planning. Based on set targets, individual and team performance was monitored daily, and daily online feedback sessions were held to address bottlenecks swiftly. The region surpassed 55% of the campaign targets for the July and September rounds. Performance against the set targets was higher during the July campaign compared to the September campaign.

Indeed, access to vaccination data is critical for evidence-based decision-making on vaccine and logistics necessities, monitoring of vaccine availability and tracking of vaccine uptake. Central to this success was careful planning, coordination and execution guided by data to identify critical gaps and inform resource allocation before and during implementation. Using facility-level data, pockets of missed populations were identified and mapped, performance among high-risk populations was monitored, and targeted interventions were implemented. In addition, geographic and socio-demographic peculiarities across councils were considered. For instance, vaccination teams delivered the vaccines to pasture camps and capitalised on events to reach nomadic communities in Kiteto. In Simanjiro, which is sparsely populated, motorcycles enabled the vaccinating teams to reach many households. Nevertheless, some limitations need to be highlighted. These findings are limited to one region in Tanzania and hence may not be representative of Tanzania or generalisable to the rest of African countries. Shortages of data collection tools and the use of manual records, which were later transferred onto electronic records, resulted in poor timeliness of reporting and could have also interfered with the completeness of the data. Despite this limitation, data entry and verification exercises were conducted after the campaigns, improving data quality and completeness.

## Conclusion

In summary, success in improving COVID-19 vaccination coverage in Manyara was achieved primarily through conducting mass vaccination campaigns. This achievement resulted from several synergistic factors: detailed planning enhanced optimal resource allocation, utilisation of data-enabled evidenced-based decision-making, and advocacy and community engagement fostered ownership at the grassroots level. This achievement resulted from several synergistic factors: detailed planning enhanced optimal resource allocation, utilisation of evidenced-based decision-making, and advocacy and community engagement fostered ownership at the grassroots level. The findings highlight the impact of the house-to-house vaccination strategy as an effective mechanism to improve COVID-19 vaccination coverage in poorly performing areas rapidly. While mass campaigns are effective and impactful, the long-term resource requirements are not sustainable. As a follow-up to the gains made from the campaigns, the need to integrate COVID-19 vaccination with routine immunisation and other essential health services becomes evident.

### What is known about this topic

- COVID-19 remains a health and socio-economic threat due to the rapid evolution of the disease, emergence of new variants and the associated impact on fragile health systems;
- Introduction of COVID-19 vaccines had a substantial impact on the evolution of the pandemic;
- COVID-19 vaccination coverage in African countries remains much lower compared to the rest of the world.

### What this study adds

- Manyara gained valuable experience in employing mixed strategies for vaccine delivery, taking into account population peculiarities across the region;
- Despite challenges with access to real time data, disaggregation of data and timely analysis to guide resource allocation and to inform strategies was effective. There is need to strengthen data management and reporting capacities to further guide decision-making;
- For sustainability and optimal resource utilisation, there is need to transition to integration of COVID-19 vaccination with routine immunisation and other Primary Health Care (PHC) services.
